# Giant liposarcoma of esophagus: a rare case report

**DOI:** 10.1186/s13000-023-01387-9

**Published:** 2023-09-05

**Authors:** Qingjiao Li, Si Chen, Yanchun Li, Zhihong Chen, Yu Liu, Wei Guo

**Affiliations:** Department of Pathology, Furong District, Hunan Provincial People’s Hospital, The First Affiliated Hospital of Hunan Normal University, Hunan Province, 61 Jiefang West Road, Changsha City, 410000 China

**Keywords:** Liposarcoma, Esophagus, Esophageal liposarcoma, Case report

## Abstract

**Background:**

Liposarcoma is a malignant mesenchymal tumor that most commonly involves the retroperitoneum and lower extremities. However, liposarcoma of esophagus has been rarely reported in the literature.

**Case presentation:**

We report a case of a 46-year-old man with complaint of intermittent dysphagia for 6 years, accompanied with paroxysmal vomiting of pedicled tumor to the mouth. Imaging studies showed a huge mixed density lesion in the middle esophageal lumen. Surgical resection of the tumor was performed through an external cervical approach. Microscopically, the tumor was composed of mature adipocytes in normal adipose tissue prominently intersected by sparsely cellular fibrous septa containing atypical, enlarged spindle cells with hyperchromatic nuclei. Immunohistochemically, the tumor cells were positive for Vimentin, S-100, CD34 and MDM2. Besides, fluorescence in situ hybridization (FISH) analysis indicated the presence of amplification involving *MDM2* gene. The patient was diagnosed as having esophageal well-differentiated liposarcoma and recovered well after the operation.

**Conclusions:**

Esophageal liposarcoma is an extremely rare tumor. Due to the nonspecific clinical manifestation and lack of experience, it is challenging to make a clear diagnosis before operation. Definite diagnosis of esophageal liposarcoma depends on histopathology, immunohistochemistry and molecular analysis.

## Background

Liposarcoma is one of the common malignant mesenchymal tumors which mostly occurs in the retroperitoneum and lower extremities [1]. However, esophageal liposarcoma is distinctly uncommon and has been rarely reported in the literature. According to the fifth edition of the World Health Organization (WHO) classification of soft tissue tumors, there are mainly five pathological subtypes of liposarcoma: well-differentiated, dedifferentiated, myxoid, pleomorphic, and myxoid pleomorphic [2]. Among them, well-differentiated liposarcoma is the most common subtype. Due to the lack of experience, it is difficult to make a clear diagnosis before operation and provide accurate and timely treatment. Imaging examinations are helpful for auxiliary diagnosis, but they have some limitations in defining the nature of the disease. The symptoms and signs of esophageal well-differentiated liposarcoma and benign esophageal tumors are similar. Both of them present as intraluminal painless masses, with smooth surface and clear boundaries or capsule [3, 4]. Therefore, it is difficult to distinguish from them in clinic and necessitate complete tumor removal. The confirmation depends on post-operative histopathology, combined with immunohistochemistry and molecular analysis.

## Case presentation

**Fig. 1:**
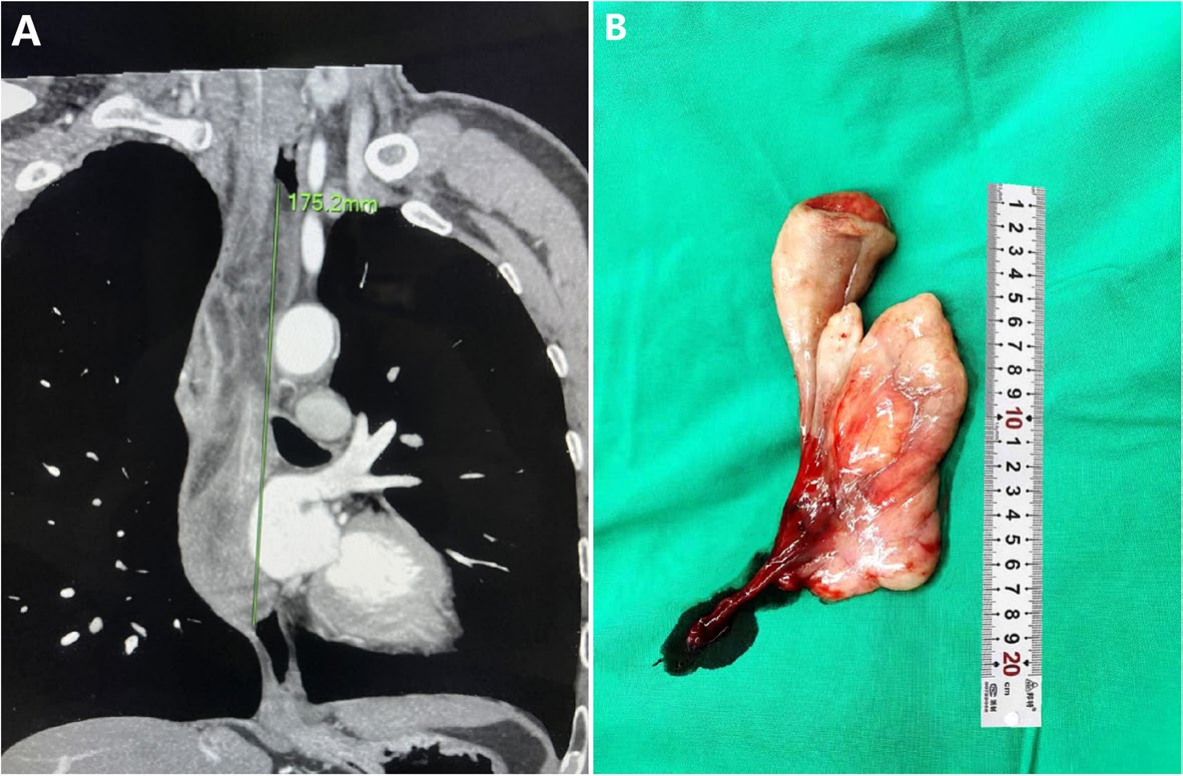
Image and surgical gross picture of the tumor. **A** Contrast-enhanced computed tomography (CT) of the chest showed a huge mixed density shadow in the middle esophageal lumen. **B** The excised specimen was a large lobulated tumor with a pedicle and smooth surface

A 46-year-old man with complaint of intermittent dysphagia of unknown origin for 6 years was admitted to our hospital. The patient found that sometimes he could extrude fleshy tissue from the mouth and was able to re-swallow it back by himself. Recently, the patient felt the symptom of dysphagia worsened, accompanied by retrosternal pain and heartburn. The medical history of the patient was not remarkable. No obvious abnormality was found by physical examination and laboratory analysis. Iodine esophagogram revealed a hill-like filling defect at the level of T6-T7 (sixth to seventh sternal vertebra) in the esophagus, accompanied by contrast reflux and disordered arrangement of mucosal folds. Contrast-enhanced computed tomography (CT) of the chest revealed a mixed density lesion in the middle esophageal lumen, with an upper and lower diameter of about 19.8 cm and a cross section of about 3.7 cm × 2.2 cm (Fig. 1A). Gastroscopy showed a large lobulated tumor could be seen from the pyriform fossa to an extent 35 cm from the incisor, occupying the esophageal cavity for 4/5 weeks, and the pedicle was located in the front wall of the entrance of the esophagus. Considering that the mass could not be removed from the esophagus, the tumor was completely removed along the pedicle through the external cervical approach under general anesthesia. Upon macroscopic examination, part of the surface of the tumor was found to be covered by normal esophageal mucosa. A large lobulated tumor, about 20 × 4 × 2.5 cm in size, with a pedicle and smooth surface could be seen (Fig. 1B).

**Fig. 2:**
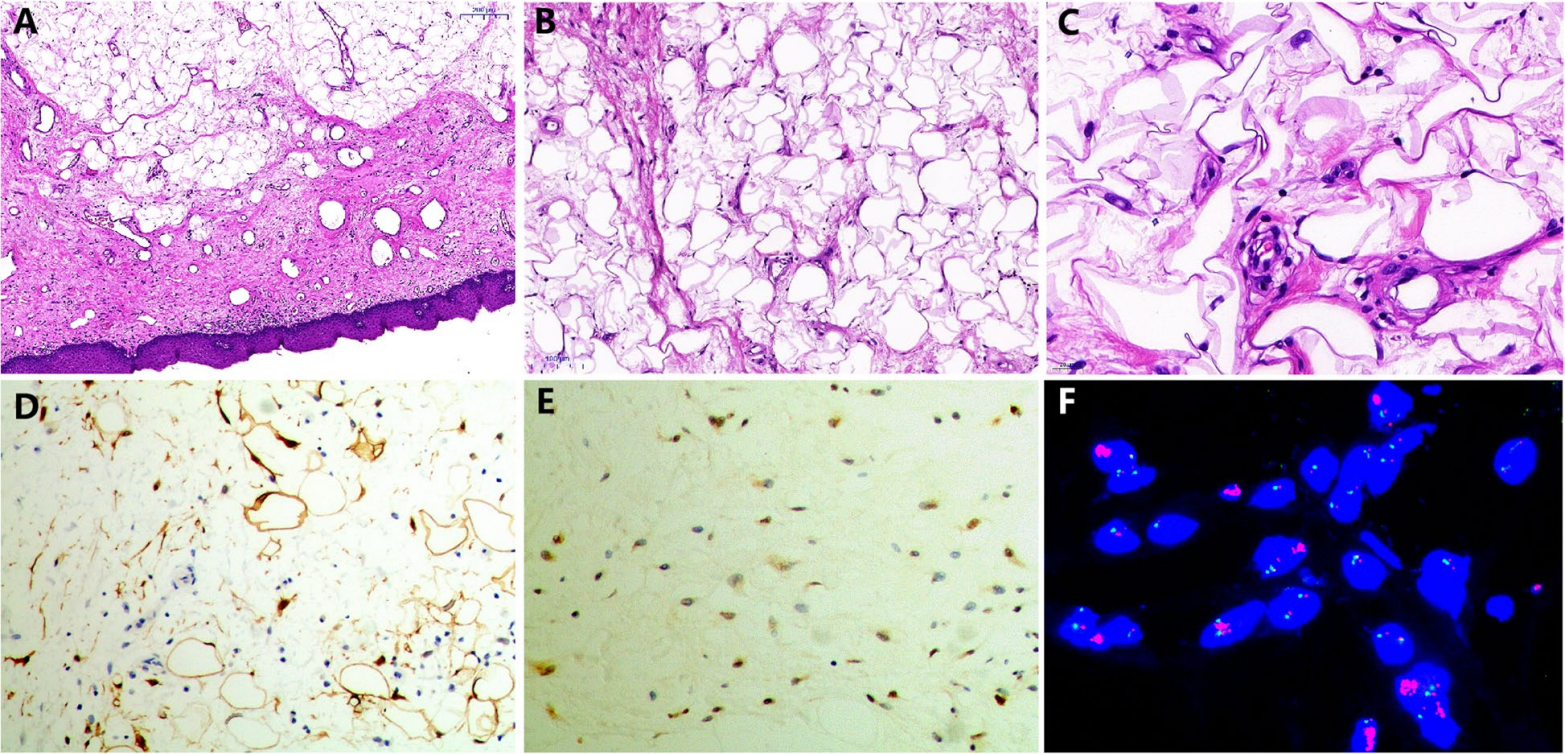
Histopathology, immunohistochemistry and molecular analysis of the tumor. **A** Histopathology analysis showed the tumor was centered in the sub-epithelial stroma and was lined by intact squamous mucosa (HE × 100). **B** & **C** The tumor was composed of mature adipocytes and a few atypical, enlarged spindle cells with hyperchromatic nuclei (B: HE × 200 and C: HE × 400). **D** & **E** Immunohistochemical staining revealed the tumor cells were positive for S-100 and MDM2 (D: HE × 200 and E: HE × 200). **F** *MDM2* gene amplification (red signal) was confirmed by fluorescence in situ hybridization (FISH) analysis

Microscopically, the tumor was centered in the sub-epithelial stroma and was lined by intact squamous mucosa (Fig. 2A). The tumor was composed of mature adipocytes in normal adipose tissue prominently intersected by sparsely cellular fibrous septa containing atypical, enlarged spindle cells with hyperchromatic nuclei (Fig. 2B & C). Immunohistochemical staining revealed the tumor cells were positive for Vimentin, S-100 (Fig. 2D), CD34 and MDM2 (Fig. 2E). The Ki-67 proliferation index was estimated to be 10%. In addition, fluorescence in situ hybridization (FISH) analysis indicated the presence of amplification involving *MDM2* gene (Fig. 2F).

Based on these findings, a diagnosis of esophageal well-differentiated liposarcoma was made. The patient was treated with fasting, anti-infection, hemostasis and rehydration, and there was no complication or local recurrence in the first 1 month of follow-up.

## Discussion and conclusions

Liposarcoma is a common malignant soft tissue tumor that mostly involves the retroperitoneum and the deep soft tissues of lower extremities [1]. However, esophageal liposarcoma is extremely rare. Mansour et al. reported the first case of esophageal liposarcoma in 1983 [5]. Up to present, less than 50 cases have been reported in the English literature [6, 7]. Based on data from the few cases available, esophageal liposarcoma tends to occur predominantly in males with a male-to-female ratio of approximately 3:1 at an average age of 58.4 years (range: 38–73 years) [8, 9]. Most esophageal liposarcoma was located in the cervical portion of the esophagus [10], while the tumor in our case was extended from the cervical esophagus down to the level of the thoracic esophagus. Due to its slow-growing nature, esophageal liposarcoma causes only minimal symptoms and tends to be large when clinically detected. Progressive dysphagia is the most common symptom, accompanied by weight loss, retrosternal pain, vomiting of the tumor into the mouth and self-ingestion [3, 11]. Some patients have dyspnea due to the huge pressure of the tumor on the respiratory tract [12]. A clear and timely diagnosis is very challenging because of the nonspecific clinical manifestations. Imaging examinations are helpful for auxiliary diagnosis. Barium meal examination, gastroscopy and endoscopic ultrasonography can better obtain the information of esophageal mucosa, esophageal peristalsis and lesion range. However, they have some limitations in defining the nature of the disease before operation. In our case, imaging findings presented as a pedicled intraluminal mass with smooth surface, which were difficult to distinguish from benign esophageal tumors including lipoma, and could easily mislead physicians to make an incorrect diagnosis. Therefore, the confirmation depends on post-operative histopathology examination. Adipocytes in different stages similar to embryonic development are the key to the pathological diagnosis of this tumor. The histopathological findings presented in our case were consistent with those described in previous reports [13, 14]. Immunohistochemistry showed the tumor cells were positive for Vimentin, S-100, CD34 and MDM2. Studied have shown that detection of *MDM2* gene amplification is the best method to distinguish well-differentiated liposarcoma from benign lipoma with sensitivity and specificity [10, 15, 16]. Significantly, *MDM2* gene amplification was confirmed by fluorescence in situ hybridization (FISH) analysis in our case. The post-operative histopathology combined with immunohistochemistry and molecular analysis confirmed the diagnosis of well-differentiated liposarcoma of esophagus.

Esophageal liposarcoma needs to be differentiated from the benign lipoma. Liposarcoma is usually large in size and composed of mature adipose tissue and irregular fibrous septa containing diagnostic atypical hyperchromatic stromal cells and less commonly lipoblasts, which are different from benign lipomas. Cases of well-differentiated liposarcoma may clinically mimic giant fibrovascular polyp [14, 17]. Atypical adipocytes and immunohistochemical expression of MDM2 should not be found in fibrovascular polyps [10]. When the fat component in the tumor decreases and more spindle cells appear, the differential diagnosis of esophageal liposarcoma also includes non-lipogenic mesenchymal tumors, such as gastrointestinal stromal tumors, schwannoma and smooth muscle tumors. Imaging examinations have demonstrated great accuracy in identifying the tumor and calculating the fat component of the tumor. The final diagnosis can be confirmed by histopathological examination. Additional immunohistochemical and molecular genetic tests, such as *MDM2* overexpression and/or amplification can be helpful for the diagnosis of liposarcoma.

Complete resection with a negative surgical margin is the core curative treatment, which can not only remove the tumor to relieve obstruction, but also achieve the goal of radical cure. Surgical approaches are diverse and include total or subtotal esophagectomy or minimally invasive approaches. With the review of literature, external cervical approach, thoracoscopic esophagotomy or endoscopic approach can be considered for surgery [3, 12, 18, 19]. Because of the unusual size and location of the tumor, we took an external cervical approach. The prognosis of esophageal liposarcoma remains unknown as the number of cases published in the literature is insufficient for rigorous analysis. Studies have showed that liposarcoma in the head and neck is usually well-differentiated, with a low grade of malignancy and better prognosis compared to liposarcoma in other sites [20]. Considering the relatively good prognosis of this disease, neck lymph node dissection and adjuvant treatment were not performed in our case. The patient recovered well after operation and there was no complication or local recurrence in the first 1 month of follow-up.

In conclusion, esophageal liposarcoma is an extremely rare tumor. Due to the nonspecific clinical manifestation and lack of experience, it is challenging to make a clear diagnosis before operation. Definite diagnosis of esophageal liposarcoma depends on histopathology, immunohistochemistry and molecular analysis. Complete surgical resection with a clear margin and a long-term follow-up after operation is essential to improve the prognosis.

## Data Availability

All data generated or analyzed during this case are included within the article.

## Abbreviations

FISH: Fluorescence in situ hybridization
CT: Computed tomography
WHO: World Health Organization
S-100: Soluble protein-100
MDM2: Murine double minute 2
CD34: Cluster of differentiation 34

## References

[1] Thway K (2019). Well-differentiated liposarcoma and dedifferentiated liposarcoma: An updated review. Semin Diagn Pathol.

[2] Choi JH, Ro JY (2021). The 2020 WHO classification of tumors of soft tissue: selected changes and new entities. Adv Anat Pathol.

[3] Ferrari D, Bernardi D, Siboni S, Lazzari V, Asti E, Bonavina L (2021). Esophageal lipoma and liposarcoma: a systematic review. World J Surg.

[4] McCarthy AJ, Carroll P, Vajpeyi R, Darling G, Chetty R (2019). Well-Differentiated Liposarcoma (Atypical Lipomatous Tumor) Presenting as an Esophageal Polyp. J Gastrointest Cancer.

[5] Mansour KA, Fritz RC, Jacobs DM, Vellios F (1983). Pedunculated liposarcoma of the esophagus: a first case report. J Thorac Cardiovasc Surg.

[6] Zhang J, Lan, Chen J, Wei X. Resection of a Giant Hypopharyngeal Liposarcoma Invading the Esophagus by Lateral Pharyngotomy: A Case Report. Ear Nose Throat J. 2020;101(9):397–402. 10.1177/0145561320973776 33179530

[7] Ben Safta Y, Souai F, Maatouk M, Zehani A, Mabrouk A, Daldoul S, Sayari S, Haout K, Ben Moussa M (2019). Myxoid esophageal liposarcoma: A case report of a rare tumor. Int J Surg Case Rep.

[8] Valiuddin HM, Barbetta A, Mungo B, Montgomery EA, Molena D (2016). Esophageal liposarcoma: Well-differentiated rhabdomyomatous type. World J Gastrointest Oncol.

[9] Lin ZC, Chang XZ, Huang XF, Zhang CL, Yu GS, Wu SY, Ye M, He JX (2015). Giant liposarcoma of the esophagus: A case report. World J Gastroenterol.

[10] Graham RP, Yasir S, Fritchie KJ, Reid MD, Greipp PT, Folpe AL (2018). Polypoid fibroadipose tumors of the esophagus: 'giant fibrovascular polyp' or liposarcoma? A clinicopathological and molecular cytogenetic study of 13 cases. Mod Pathol.

[11] Dowli A, Mattar A, Mashimo H, Huang Q, Cohen D, Fisichella PM, Lebenthal A (2014). A pedunculated giant esophageal liposarcoma: a case report and literature review. J Gastrointest Surg.

[12] Takiguchi G, Nakamura T, Otowa Y, Tomono A, Kanaji S, Oshikiri T, Suzuki S, Ishida T, Kakeji Y (2016). Successful resection of giant esophageal liposarcoma by endoscopic submucosal dissection combined with surgical retrieval: a case report and literature review. Surg Case Rep.

[13] Fritchie K, Ghosh T, Graham RP, Roden AC, Schembri-Wismayer D, Folpe A, Rivera M (2020). Well-Differentiated/Dedifferentiated Liposarcoma Arising in the Upper Aerodigestive Tract: 8 Cases Mimicking Non-adipocytic Lesions. Head Neck Pathol.

[14] Mehdorn AS, Schmidt F, Steinestel K, Wardelmann E, Greulich B, Palmes D, Senninger N (2017). Pedunculated, well differentiated liposarcoma of the oesophagus mimicking giant fibrovascular polyp. Ann R Coll Surg Engl.

[15] Myung JK, Jeong JB, Han D, Song CS, Moon HJ, Kim YA, Kim JE, Byun SJ, Kim WH, Chang MS (2011). Well-differentiated liposarcoma of the oesophagus: clinicopathological, immunohistochemical and array CGH analysis. Pathol Oncol Res.

[16] Chen C, He X, Jing WY, Qiu Y, Chen M, Luo TY, Liu XY, Chen HJ, Zhang HY, Bu H (2022). Diagnostic value of MDM2 RNA in situ hybridization in atypical lipomatous tumor/well-differentiated liposarcoma and dedifferentiated liposarcoma. Zhonghua Bing Li Xue Za Zhi.

[17] Jakowski JD, Wakely PE (2009). Rhabdomyomatous well-differentiated liposarcoma arising in giant fibrovascular polyp of the esophagus. Ann Diagn Pathol.

[18] Ye YW, Liao MY, Mou ZM, Shi XX, Xie YC (2020). Thoracoscopic resection of a huge esophageal dedifferentiated liposarcoma: A case report. World J Clin Cases.

[19] Bernardi D, Ferrari D, Siboni S, Porta M, Bruni B, Bonavina L (2020). Minimally invasive approach to esophageal lipoma. J Surg Case Rep.

[20] Gerry D, Fox NF, Spruill LS, Lentsch EJ (2014). Liposarcoma of the head and neck: analysis of 318 cases with comparison to non-head and neck sites. Head Neck.

